# Prevalence of neurocognitive impairments in adults with chronic pain: A cross-sectional study

**DOI:** 10.4102/sajpsychiatry.v31i0.2500

**Published:** 2025-09-25

**Authors:** Bonginkosi M.J. Mafuze, Lindokuhle T. Thela

**Affiliations:** 1 Department of Psychiatry, College of Health Sciences, University of KwaZulu-Natal, Durban, South Africa

**Keywords:** chronic pain, neurocognitive impairments, MoCA, MMSE, prevalence

## Abstract

**Background:**

There is a strong link between chronic pain and neurocognitive impairment. The co-occurrence of the two disorders often leads to a poor quality of life and significant disability.

**Aim:**

To determine the prevalence of neurocognitive impairments in adults with chronic pain.

**Setting:**

The study was conducted at a tertiary hospital in Pietermaritzburg, KwaZulu-Natal.

**Methods:**

This cross-sectional study was conducted at a pain clinic within a tertiary hospital in Pietermaritzburg, KwaZulu-Natal. Participants were required to be clinic attendees, proficient in English, and have a minimum of a Grade 7 education. Exclusion criteria included neurological disorders, significant language barriers, or ineligible age. Recruitment used purposive sampling with informed consent. Data were collected using socio-demographic and clinical questionnaires, namely, the Mini-Mental State Examination (MMSE), the Montreal Cognitive Assessment (MoCA), and the Physical Self-Maintenance Scale (PSMS). The primary outcome was the prevalence of neurocognitive impairment; secondary outcomes examined associations with demographic and clinical factors using both descriptive and inferential statistics.

**Results:**

A total of 105 participants (59 females and 46 males) were enrolled in the study. The mean age of the participants was 55.1 ± 6.75 years. A total of 73.3% (*n* = 77) of participants screened positive for neurocognitive impairment on MoCA and 55.2% on MMSE.

**Conclusion:**

Chronic pain is associated with impairments in neurocognitive performance, particularly in short-term memory and executive functioning.

**Contribution:**

A significant correlation was found between lower education levels and neurocognitive impairment (*p* = 0.02 for MoCA, *p* = 0.04 for MMSE).

## Introduction

Chronic pain is a debilitating condition that is linked with disability.^1^ It has been estimated that 20% of adults worldwide experience chronic pain, with a higher prevalence of 33% observed in low- and middle-income countries (LMICs).^2,3^ One in five adults in South Africa experiences chronic pain.^4^ On a global scale, chronic pain, particularly lower back pain, represents the primary cause of years lived with disability (YLDs).^5^ Chronic pain is frequently linked to impairments across multiple neurocognitive domains, including attention, memory, learning, processing, problem-solving, and motor function.^6,7,8^

More than 50% of individuals suffering from chronic pain demonstrate cognitive impairment.^1,9,10^ Many people face this dual burden without the care or treatment needed to manage their pain and cognitive issues.^11^ Chronic pain patients often face neurocognitive challenges that impair functioning, quality of life, treatment adherence, and mental health.^12^ A clear understanding of how common neurocognitive impairments are in individuals with chronic pain can help guide better care strategies.^13^ Chronic pain causes physical, emotional, financial, and social burdens for patients, caregivers, and society.^14,15^

Healthcare professionals can create personalised plans addressing pain and cognitive issues, improving the quality of life for affected individuals.^16^ Identifying neurocognitive impairments can help healthcare systems implement targeted interventions, reduce costs, and improve resource allocation.^17^ Numerous factors contribute to the inadequate assessment of neurocognitive deficits in patients experiencing chronic pain. Research indicates that subjective cognitive complaints may be associated with depressive symptoms.^18^ Alternative factors may include restricted availability of neuropsychological testing resources, time limitations, and the requisite expertise for administering these assessments.^19^ Some studies have identified the existence of complex patterns and correlations between chronic pain conditions and the presence of neurocognitive impairments.^20^ Therefore, this type of study is crucial for enhancing our understanding of the burden of chronic pain on cognitive function in this setting.^8^

## Research methods and design

### Study design

This was a cross-sectional, quantitative, observational study conducted from January 2023 to July 2023 at the Greys Hospital Tertiary Pain Clinic in Pietermaritzburg, KwaZulu-Natal, South Africa. The clinic serves a large, diverse population from urban, peri-urban, and rural areas, with the majority of patients being referred from primary or district-level facilities within the public health system. The multidisciplinary team includes anaesthesiologists, psychiatrists, psychologists, physiotherapists, occupational therapists, pharmacists, nurses, and social workers. The study sample comprised adult clinic attendees presenting for assessment or follow-up, predominantly black South Africans with varied educational backgrounds and a high prevalence of comorbid physical and mental health conditions, broadly representative of the provincial chronic pain population.

### Study population and sampling strategy

Consecutive adult patients attending their routine follow-up appointments at the Greys Hospital Tertiary Pain Clinic (Wednesdays and Fridays) between January 2023 and July 2023 were invited to participate. Consecutive sampling was used to minimise selection bias and enhance the representativeness of the clinical population. Patients are typically referred from district hospitals and community health centres across KwaZulu-Natal for multidisciplinary pain management. Inclusion criteria were: age 18–65, a confirmed diagnosis of chronic pain (≥ 3 months), completion of initial rehabilitation, proficiency in English (reading, writing, and speaking), and a minimum of Grade 7 education. Exclusion criteria included known neurological disorders, active psychosis, or significant communication impairments. Over the study period, 121 consecutive patients were seen; of these, 105 met eligibility criteria and were enrolled. The population was demographically mixed, predominantly black South African, with musculoskeletal pain, post-traumatic pain, and mixed neuropathic syndromes being the most common diagnoses. Study information sheets and flyers were distributed in the clinic waiting area.

### Statistics and sample size justification

Sample size estimation was based on a 20% prevalence of cognitive impairment reported by Landro et al.^21^ In the cohort of patients experiencing chronic pain, a sample size of 110 participants was required to achieve a 95% confidence interval with a margin of error of 7.5%, considering a 10% non-response rate; the final sample size to be achieved was 121 participants.

### Data collection

The principal investigator collected all data. A clinical data sheet was used to document the socio-demographic details, lifestyle factors, and clinical history. This clinical data sheet was derived from the research conducted by Narsi et al.^22^ All data were collected during a single clinic visit, taking approximately 25–30 min to administer. Each participant was first assessed with the Mini-Mental State Examination (MMSE), then the Montreal Cognitive Assessment (MoCA). The tests were administered and scored as instructed by the relevant instrument.

### Study measures/instruments

#### The Mini-Mental State Examination

The MMSE was developed by Folstein et al. specifically as a screening instrument for Alzheimer’s dementia,^23^ and has also been used to assess a range of other neurodegenerative and neurocognitive disorders. This examination serves as a clinician-initiated cognitive assessment tool utilised to evaluate cognitive function across various domains, including Orientation, Registration, Attention and Concentration, Memory and Language, as well as Visuospatial capabilities. The maximum achievable score is 30, and within the framework of this study, a normal cut-off score of 24 or above is considered indicative of normal cognitive function. Conversely, a score below 24 is categorised as abnormal, indicating possible cognitive impairment. The MMSE has been used in South African populations in a study by Mfene et al. in KwaZulu-Natal.^24^ The Cronbach’s alpha score for the MMSE was 0.703, suggesting that the MMSE has acceptable internal consistency in this specific study population.

#### The Montreal Cognitive Assessment

Montreal Cognitive Assessment is a succinct cognitive screening tool that exhibits heightened sensitivity and specificity in detecting mild cognitive impairment, Most individuals meeting the clinical criteria for MCI (Mild cognitive impairment) score above 26 (within the normal range) on the MMSE. For the purposes of this study, a score below 26 out of 30 has been considered abnormal. The MoCA has been validated in the South African population before in a study by Beath et al.^25^ The Cronbach’s alpha score for the MoCA was reported as 0.64, indicating moderate internal consistency. The study also found that MoCA scores were significantly correlated with education and age, but not with gender.

#### The Physical Self-Maintenance Scale

The Physical Self-Maintenance Scale (PSMS) is a functional assessment tool that evaluates an individual’s basic activities of daily living (ADLs). It assesses physical functioning and independence in performing essential self-care tasks, such as feeding, dressing, toileting, and mobility. It is particularly useful in identifying functional impairments in older adults and individuals with chronic illnesses. The PSMS assesses physical self-maintenance through six self-care activities, each scored from 1 to 5. Total scores range from 6 (fully independent) to 30 (fully dependent), with lower scores indicating better functional ability. Physical Self-Maintenance Scale has been used to assess physical functioning and independence in chronic illness, including chronic pain and older adults with multiple co-morbidity in similar populations and low-middle income settings.^26,27,28^ The PSMS has good inter-rater reliability of 0.87;^29^ it is also a validated tool used by Esaadi M et al. in a similar patient cohort in KwaZulu-Natal.^30^

### Data analysis

The data were systematically documented within a password-protected REDCap mobile database. Statistical analysis was conducted utilising Stata version 17, wherein Chi-Square tests or Fisher’s exact tests were employed for categorical variables, and *t*-tests or Mann-Whitney U tests were applied for continuous variables, contingent upon the data distribution. The normality of continuous data was assessed using the Shapiro-Wilk test, with the data presented as means alongside standard deviations (for normally distributed variables) or medians accompanied by interquartile ranges (for non-normally distributed variables). Categorical data were summarised as frequencies and percentages. To mitigate the impact of potential confounding variables, a multivariate analysis was performed, adjusting for pertinent socio-demographic and clinical factors. Associations between cognitive performance and independent variables, such as education level and cognitive impairment, were analysed using logistic regression models, establishing statistical significance at *p* < 0.05.

### Ethical considerations

Approval for the access of medical records was acquired from the KwaZulu-Natal (KZN) Department of Health and the ethics committee of Greys Hospital prior to the initiation of data collection. Ethical clearance to conduct the study was granted by the Biomedical Research Ethics Committee of the University of KZN (reference number BREC/00004986/2022).

## Results

A total of 121 participants were recruited from the outpatient department of the pain clinic. During the study period, 16 participants were excluded because of age limitations and language barriers. As a result, 105 participants (46 males vs. 59 females) were included in the final analysis. None of the participants had a diagnosed psychotic disorder or exhibited symptoms of a formal thought disorder that would impair their ability to communicate or participate in the assessments. All participants demonstrated satisfactory cooperation. Participants’ demographic data are shown in Table 1.

**TABLE 1:** Socio-demographic profile of study participants with chronic pain (*N* = 105).

Demographic	Variable	*n*	%
Age (years)			
	Range	32–65 years	-
	Mean	55.11 ± 6.75 years	-
	Median	56 years	-
Gender			
	Male	46	43.8
	Female	59	56.2
Ethnicity			
	Black people	61	58.1
	Indian people	27	25.7
	Mixed race	7	6.7
	White people	10	9.5
Highest level of education			
	**Secondary (non-tertiary):** Grade 7 – Grade 12	**47**	**44.8**
	**Tertiary:**	**58**	**55.2**
	FET	24	22.9
	Technikon	23	21.9
	University	10	9.5
	Other	1	0.9
Home language			
	IsiZulu	58	55.2
	English	45	42.9
	Other	2	1.9
Employment			
	Yes	14	13.3
	No	91	86.7

FET, Further Education and Training.

The socio-demographic profile of participants experiencing chronic pain indicates a mean age of 55 years. Notable associations were found concerning gender and neurocognitive impairment (43.8% male, *p* = 0.03), ethnicity (58.1% black people, *p* < 0.001), and education level (44.8% secondary, *p* = 0.04). In contrast, home language and employment status did not demonstrate any significance.

### Association between neurocognitive impairment and socio-demographic factors

The analysis revealed no significant correlation between the presence of neurocognitive impairment, as assessed, and age. However, the male gender, evaluated through the MMSE, exhibited clinical significance (*p* = 0.03). In addition, individuals of African descent, evaluated by the MoCA were also found to be clinically significant (*p* = 0.001). The primary language spoken at participants’ residences did not demonstrate any clinical significance. Furthermore, individuals with no tertiary education showed a significant association with neurocognitive impairment, as indicated by both MMSE (*p* = 0.04) and MoCA (*p* = 0.02). The employment status of participants had no effect on the results of the assessments administered.

The clinical characteristic data presented in Table 2 indicate that 18% (*n* = 19) of the participants experienced a comorbid mental health condition, whereas 89.5% (*n* = 94) reported the presence of at least one medical comorbidity. None of the participants reported a history of head trauma or alcohol abuse; however, a significant 68.6% (*n* = 72) indicated a lifetime history of substance use. Importantly, back pain was identified as the most frequently reported type of chronic pain, with 93 individuals (88.6%) acknowledging its presence.

**TABLE 2:** Clinical characteristics of study participants with chronic pain.

Characteristic	Outcome	*n*	%
Presence of any mental illness	Yes	19	18.1
	No	86	81.9
Presence of any medical co-morbidity	Yes	94	89.5
	No	11	10.5
Lifetime use of any substance	Yes	72	68.6
	No	33	31.4

The clinical characteristics of participants with chronic pain showed no significant associations with any known mental illness diagnosis, medical comorbidity, or lifetime use of any substance. However, the MoCA alone did indicate that the presence of mental illness is significant with *p* < 0.05.

**FIGURE 1:**
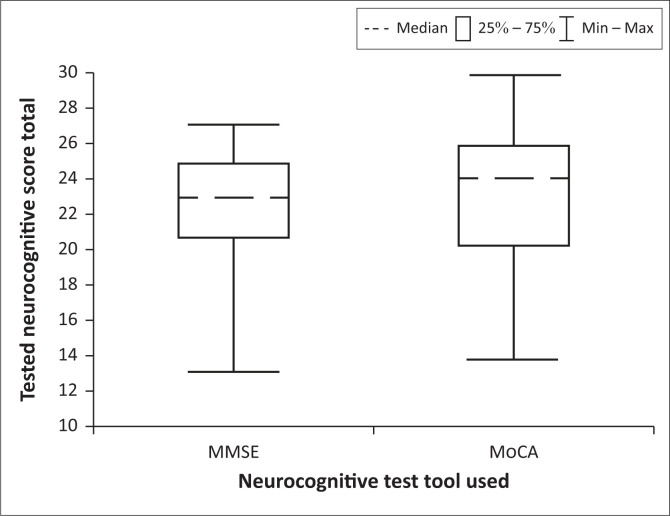
Neurocognitive testing scores administered to the study participants.

The prevalence of neurocognitive impairment among the evaluated participants was determined to be 55.2% (*n* = 58) based on the MMSE, and 73.3% (*n* = 77) as indicated by the MoCA. The statistical results are graphically presented in Figure 1.

The box and whisker plot for MMSE scores shows a minimum score of 13 and a maximum score of 27, with an interquartile range (IQR) from 21 to 25 and a median of 23. For MoCA scores, the plot indicates a minimum score of 14 and a maximum score of 30, with an IQR from 20 to 26 and a median score of 24.

### Association of neurocognitive impairment and clinical factors

**FIGURE 2:**
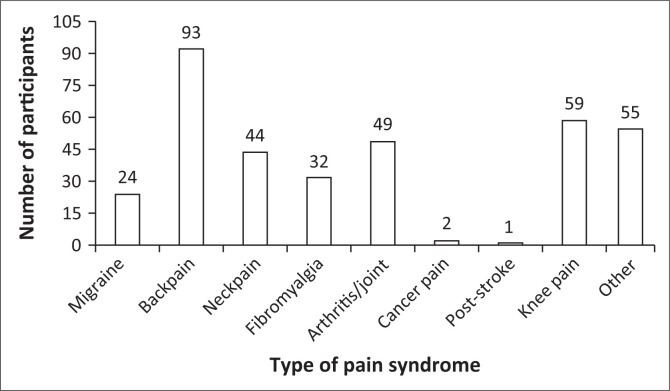
Graph showing the pain syndrome types and number of participants affected.

Participants who presented with medical comorbidities and a history of lifetime substance use did not demonstrate any statistically significant findings. The use of the MoCA indicated a significant correlation with the presence of a mental illness (*p* = 0.005). In conclusion, the type of pain was not significantly associated with any impairment in neurocognition. Figure 2 illustrates the type of pain syndrome and the number of participants affected.

The bar graph shows back pain as the most prevalent pain syndrome, reported by 93 of 105 participants, with many experiencing multiple co-occurring pain syndromes; ‘Other’ includes conditions like Complex Regional Pain Syndrome (CRPS), hand and/or shoulder pain, post-operative pain, and others.

## Discussion

The study demonstrated a significant prevalence of neurocognitive impairment among the participants, with variations in results dependent on the assessment instruments used. Certain socio-demographic factors, such as male gender, African ethnicity, and a lack of tertiary education, exhibited noteworthy correlations with neurocognitive impairment. In contrast, employment status and medical comorbidities did not reveal any considerable influence; however, the presence of a mental illness was significantly associated with neurocognitive impairment as assessed by the MoCA. Furthermore, the type of chronic pain and lifetime substance use did not show any significant correlations with the outcomes of cognitive impairment.

The primary findings correspond with established research regarding the intricate relationship between chronic pain, cognitive function, and sociodemographic factors.^31^ The high prevalence of neurocognitive impairment in this sample supports previous research, showing that chronic pain often causes cognitive deficits.^9^ This phenomenon may be attributable to shared neural mechanisms encompassing pain processing and cognitive functions.^7,32,33^ The discrepancy in impairment rates between the MMSE and the MoCA is consistent with existing literature, which indicates that the MoCA demonstrates greater sensitivity in detecting mild cognitive impairment dysfunction.^34,35,36,37^ This study shows identical medians in both tests but differing cut-off scores and IQR distributions, all within mild cognitive impairment. This suggests that at least 50% of the sample with chronic pain exhibited neurocognitive impairment, which is in keeping with some research findings.^7^

The correlation between neurocognitive impairment and factors such as gender, ethnicity, and education aligns with research on social health determinants that may impact cognitive outcomes.^38,39^ Previous studies have indicated that lower levels of education may diminish cognitive abilities reserve,^40,41,42^ rendering individuals increasingly susceptible to impairment. The absence of a correlation between employment status and impairment contrasts with studies suggesting unemployment negatively affects cognitive function,^43,44,45,46^ indicating the necessity for additional inquiry in this matter.

The strong correlation between mental illness and cognitive impairment aligns with evidence showing mental health conditions exacerbate cognitive dysfunction in chronic pain.^9,47,48^ This underscores the significance of integrated care strategies that address cognitive and psychological dimensions in chronic pain management.

These findings underscore the necessity for targeted cognitive screening within populations experiencing chronic pain, particularly among groups identified as high-risk. The identification of a deficiency in tertiary education as a predictor of cognitive impairment suggests the potential benefits of implementing cognitive training or educational interventions to promote cognitive resilience. These results may inform healthcare strategies designed to identify and support individuals at risk of neurocognitive impairment, thereby enhancing patient outcomes in the management of chronic pain.

### Strengths and limitations

The study’s strengths include its prospective design and use of validated MMSE and MoCA for comprehensive neurocognitive evaluation. The MoCA enhances the reliability of the findings because of its heightened sensitivity in detecting mild dysfunction. The diverse sample of age, gender, and ethnicity enhances the generalisability of the results to similar clinical populations. Focusing on chronic pain patients, the study highlights the often-overlooked cognitive function, offering insights for future screening and interventions. The study considered socio-demographic and clinical factors, including education, employment, mental health, and substance use, for a holistic understanding.

Several limitations of this study are evident, particularly its cross-sectional design, which captures data at a singular point in time. This characteristic restricts the ability to establish causality between chronic pain, neurocognitive impairment, and associated factors. Recruiting from a single pain clinic may introduce selection bias, limiting generalisability and reflecting enhanced health-seeking behaviours. The study analysed cognitive function factors, but did not fully account for confounders such as medication use, pain severity, and socioeconomic status. Excluding non-native speakers because of language barriers may limit the representativeness of findings, particularly in multilingual populations. Reliance on self-reported data for substance use and comorbidities raises concerns about recall bias and underreporting. Although a correlation between neurocognitive impairment and a lack of tertiary education was found, cognitive reserve and lifelong learning were not explored.

Despite methodological limitations, this study provides valuable insights into the prevalence and associations of neurocognitive impairment in chronic pain patients. Future research with longitudinal designs and diverse samples could strengthen these findings and guide interventions to reduce cognitive decline. This study highlights the need for routine cognitive screening in chronic pain management, especially for high-risk groups such as males, Africans, and those with lower education levels.

Clinicians are encouraged to adopt a multidisciplinary methodology that integrates cognitive rehabilitation and mental health support alongside pain treatment protocols. Policy initiatives should focus on expanding education-based cognitive resilience programmes and ensuring integrated mental health and pain management services. Furthermore, efforts should be made to enhance the inclusivity of cognitive assessments by addressing language barriers. Future research should explore the causal relationship between chronic pain and cognitive impairment, considering pain severity, medication, and cognitive reserve.

Exploring the intersectionality of socio-demographic factors could yield deeper insights into cognitive outcomes among chronic pain populations. These findings highlight the importance of a holistic pain management approach, addressing cognitive, psychological, and social factors to improve outcomes.

## Conclusion

This study has provided significant insights into the demographic profile of patients attending a pain clinic. It reveals a predominantly middle-aged and unemployed population, which is characterised by a high prevalence of African ethnicity and male gender, as well as a lack of tertiary education as risk factors. Findings show significant neurocognitive impairment prevalence, differing by assessment tool – 55.2% (MMSE) and 73.3% (MoCA), emphasising nuanced screening. Several risk factors for neurocognitive impairment were identified, including male gender, African ethnicity, and lower education levels. In contrast, employment status, medical comorbidities, and substance use history exhibited no significant association. Importantly, the presence of a mental illness was significantly correlated with cognitive impairment, reinforcing the importance of integrating mental health and cognitive screening within pain management strategies. These findings highlight the necessity for targeted interventions aimed at supporting cognitive function in chronic pain patients, particularly among high-risk populations, to enhance overall health outcomes.
